# Supplementary material for: Polyketide synthase gene clusters in the ericoid mycorrhizal fungus Oidiodendron maius: genome-wide prediction, expression profiling, and functional characterization of knockout mutants
Source: Front Plant Sci. 2026 Sep 16;17:1924430. doi: 10.3389/fpls.2026.1924430 (PMC13623903; doi:10.3389/fpls.2026.1924430)
Supplement: Supplementary file 1 [file SupplementaryFile1.zip › Supplementary Materials/Supplementary Folder 1.zip!/Oidma1_AssemblyScaffolds_Repeatmasked/knownclusterblast/cluster12/jgi.p_Oidma1_116513_mibig_hits.html]

| MIBiG Protein | Description | MIBiG Cluster | MiBiG Product | % ID | % Coverage | BLAST Score | E-value |
| --- | --- | --- | --- | --- | --- | --- | --- |
| AIG62134.1 | glucose-methanol-choline\_oxidoreductase | BGC0000120 | Patulin\_biosynthetic\_gene\_cluster | 44 | 97.8512396694 | 438 | 1.3e-122 |
| AAS90019.1 | VBS | BGC0000007 | Aflatoxin\_biosynthetic\_gene\_cluster | 40 | 92.8925619835 | 375 | 1.3e-103 |
| ACH72898.1 | AflK | BGC0000011 | Aflatoxin/sterigmatocystin\_biosynthetic\_gene\_cluster | 39 | 93.3884297521 | 373 | 3.8e-103 |
| AAS90066.1 | VBS | BGC0000009 | Aflatoxin\_biosynthetic\_gene\_cluster | 39 | 92.8925619835 | 373 | 5e-103 |
| AAS90088.1 | VBS | BGC0000010 | Aflatoxin\_biosynthetic\_gene\_cluster | 40 | 92.8925619835 | 372 | 8.6e-103 |
| BAE71331.1 | versicolorin\_B\_synthase | BGC0000004 | Aflatoxin\_biosynthetic\_gene\_cluster | 40 | 92.8925619835 | 371 | 2.5e-102 |
| AAS90042.1 | VBS | BGC0000008 | Aflatoxin\_biosynthetic\_gene\_cluster | 39 | 92.7272727273 | 370 | 4.2e-102 |
| AAS90106.1 | VBS | BGC0000006 | Aflatoxin\_biosynthetic\_gene\_cluster | 40 | 92.8925619835 | 369 | 5.5e-102 |
| EAU32818.1 | predicted\_protein | BGC0000160 | Terreic\_acid\_biosynthetic\_gene\_cluster | 38 | 92.2314049587 | 351 | 2.7e-96 |
| ALI92648.1 | CitC\_oxidoreductase | BGC0001338 | Citrinin\_biosynthetic\_gene\_cluster | 28 | 98.1818181818 | 183 | 9.6e-46 |
| ACA34720.1 | CtnD | BGC0000894 | Citrinin\_biosynthetic\_gene\_cluster | 28 | 98.1818181818 | 183 | 9.6e-46 |
| ABY65992.1 | alcohol\_dehydrogenase | BGC0001008 | Maduropeptin\_biosynthetic\_gene\_cluster | 29 | 93.3884297521 | 180 | 4.8e-45 |
| ctg1\_orf10 |  | BGC0000846 | Tabtoxin\_biosynthetic\_gene\_cluster | 29 | 91.5702479339 | 176 | 6.9e-44 |
| AEF33092.1 | choline\_dehydrogenase | BGC0001039 | Pyridomycin\_biosynthetic\_gene\_cluster | 28 | 91.7355371901 | 160 | 5.1e-39 |
| BAQ25461.1 | putative\_dehydrogenase | BGC0001264 | Betaenone\_A\_/\_betaenone\_B\_/\_betaenone\_C\_biosynthetic\_gene\_cluster | 26 | 98.1818181818 | 158 | 3.3e-38 |
| CBF73676.1 | conserved\_hypothetical\_protein | BGC0001271\_c3 | Emericellin\_biosynthetic\_gene\_cluster | 31 | 56.8595041322 | 137 | 4.6e-32 |
| AJI44177.1 | glucose-methanol-choline\_oxidoreductase | BGC0001193 | Thiolutin\_biosynthetic\_gene\_cluster | 28 | 53.3884297521 | 73 | 1.1e-12 |
